# Supplementary material for: Identification of Freezing-Responsive microRNAs and Their Targets in Chinese Jujube by Small RNA and Degradome Sequencing
Source: Int J Mol Sci. 2026 Sep 4;27(17):7912. doi: 10.3390/ijms27177912 (PMC13566890; doi:10.3390/ijms27177912)
Supplement: Supplementary file 1 [file ijms-27-07912-s001.zip › Supplementary Material S2/novel_miR_195.pdf]

Provisional ID	:	novel_miR_195
Score total	:	0.3
Score for star read(s)	:	-1.3
Score for read counts	:	0
Score for mfe	:	0
Score for randfold	:	1.6
Score for cons. seed	:	
Total read count	:	622
Mature read count	:	622
Loop read count	:	0
Star read count	:	0

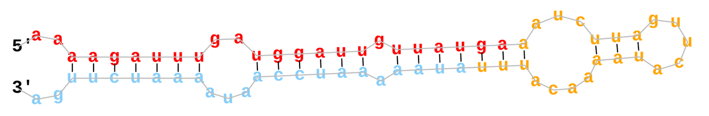

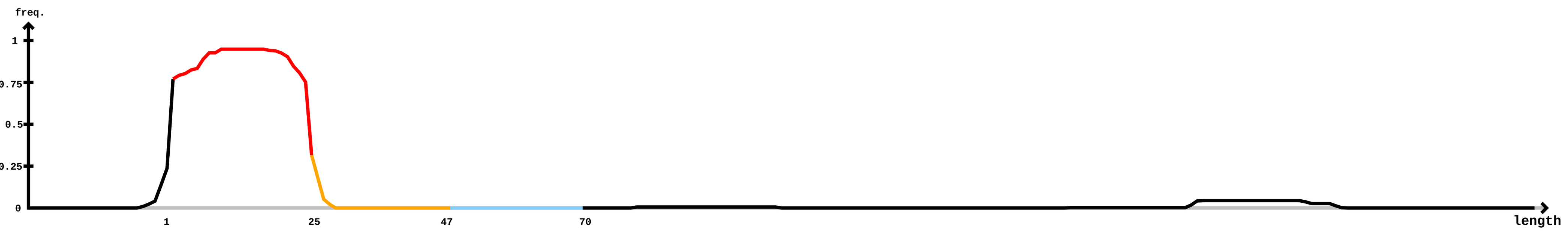

Mature **Star**

```
5'- uuuaaaaugaguuauaaacuuuaaaagauuugauggauuguuaugaaaucuuaguucauaaaacauuuauaaaaauccaauaaaaucuugaagugaagauuggauuucguauuuacaauuuucuucacaauuuuauuuuaaaauccuuucaaauauauuaaaauucauuauuuuuaaaaucuuuuaaaauuaaugaccuuuugaauaccuauagauuuuaaaauaauuuuauaaaauuauaauugaaua -3'   exp
    .......(.(((((((((((((..((((((..((((((.(((((((...(((.....)))....))))))).))))))...))))))))))))((((((((............)))))))).....((..((((((((((((........(((.((((((.((((((..(((((((....))))))).))))))..)))))))))......))))))))))))..))......))))))))).)...   reads   mm   sample
    ................acuuuaaaagauuugauggauuguu..................................................   1   0   JCK-3
    ................acuuuaaaagauuugauggauuguu..................................................   1   0   JCK-1
    ................acuuuaaaagauuugauggauuguu..................................................   3   0   JCK-2
    ................acuuuaaaCgauuugauggauuguu..................................................   1   1   JCK-3
    .................cuuuaaaagauuugauggau......................................................   5   0   LT-2
    .................cuuuGaaagauuugauggauugu...................................................   2   1   LT-1
    .................cuuuaaaagauuugauggauuguu..................................................   1   0   JCK-2
    .................cuuuaaaagauuugauggauuguu..................................................   1   0   JCK-3
    .................cuuuaaaagauuugauggauuguu..................................................   1   0   JCK-1
    ..................uuuaaaagauuugauggauuguu..................................................   1   0   LT-3
    ..................uuuaaaagauuugauggauuguu..................................................   1   0   JCK-2
    ..................uuuaaaagauuugauggauuguu..................................................   2   0   LT-2
    ..................uuuaaaagauuugauggauuguA..................................................   1   1   LT-2
    ..................uuuaaaagauuugauggauuguu..................................................   2   0   JCK-3
    ..................uuuaaaagauuugauggauuguu..................................................   2   0   JCK-1
    ..................uuAaaaagauuugauggauuguua.................................................   1   1   JCK-1
    ..................uuAaaaagauuugauggauuguua.................................................   1   1   JCK-2
    ..................uCuaaaagauuugauggauuguuaugaa.............................................   1   1   JCK-2
    ...................uuaaaagauuugauggauu.....................................................   1   0   JCK-1
    ...................uuaaaagauuugauggauu.....................................................   1   0   JCK-3
    ...................uuaaaagauuugauggauug....................................................   2   0   LT-3
    ...................uuaaaagauuugauggauug....................................................   1   0   LT-1
    ...................uuaaaagauuugauggauug....................................................   1   0   JCK-3
    ...................uuaaaUgauuugauggauugu...................................................   1   1   JCK-3
    ...................uuaaaagauuugauggauuguu..................................................   7   0   LCK-3
    ...................uuaaaagauuugauggauuguu..................................................   1   0   JCK-3
    ...................uuaaaagauuugauggauuguu..................................................   1   0   LT-1
    ...................uuaaaagauuugauggauuguC..................................................   1   1   LT-1
    ...................uuaaaagauuugauggauuguu..................................................   1   0   LT-3
    ...................uuaaaagauuugauggauuguuC.................................................   1   1   JCK-2
    ...................uuaaaagauuugauggauuguua.................................................   2   0   JCK-1
    ...................uuaaaagauuugauggauuguuU.................................................   3   1   LT-1
    ...................uuaaaagauuugauggauuguua.................................................   2   0   JCK-2
    ...................uuaaaagauuugauggauuguuC.................................................   4   1   JCK-1
```

```
                      Mature                                          Star
uuuaaaaugaguuauaaacuuuaaaagauuugauggauuguuaugaaaucuuaguucauaaaacauuuauaaaaaauccaauaaaaucuugaagugaagauuggauuucguauuuacaauuuucuucacaauuuuauuuuaaaauccuuucaaauauauuaaaauucauuauuuuuuaaaaucuuuuaaaauuaaugaccuuuugaauaccuauagauuuuaaaauaauuuuauaaaauuauaauugaaua
....................uuaaaagauuugauggauuguuG....     1    1    JCK-3
....................uuaaaagauuugauggauuguua....     4    0    JCK-3
....................uuaaaagauuugauggauuguuC....     1    1    JCK-3
....................uuaaaagauuugauggauuguuUu....    4    1    LT-2
....................uuaaaagauuugauggauuguuau....    5    0    JCK-1
....................uuaaaagauuugauggauuguuau....    4    0    JCK-3
....................uuaaaagauuugauggauuguuau....    5    0    LT-1
....................uuaaaagauuugauggauuguuau....    8    0    JCK-2
....................uuaaaagauuugauggauuguuauU....   1    1    LT-1
....................uuaaaagauuugauggauuguuaug....   1    0    JCK-3
....................uuaaaagauuugauggauuguuaug....   1    0    JCK-1
....................uuaaaagauuugauggauuguuauga....  1    0    JCK-1
.....................Aaaaagauuugauggauugu....       1    1    JCK-1
.....................Aaaaagauuugauggauugu....       1    1    JCK-3
.....................uaaaagauuugauggauuAu....       1    1    LT-1
.....................Aaaaagauuugauggauuguuaug....   2    1    JT-2
.....................Aaaaagauuugauggauuguuaug....   7    1    JCK-1
.....................Aaaaagauuugauggauuguuaug....   1    1    LT-2
.....................Aaaaagauuugauggauuguuaug....   2    1    JT-3
.....................Aaaaagauuugauggauuguuaug....   9    1    LT-3
.....................uaaaagauuugauggauuguuaug....   1    0    LT-1
.....................Aaaaagauuugauggauuguuaug....   16   1    JCK-3
.....................uUaaagauuugauggauuguuaug....   1    1    LT-1
.....................Aaaaagauuugauggauuguuaug....   19   1    JCK-2
.....................Aaaaagauuugauggauuguuaug....   4    1    LT-1
.....................uaaaagauuugauggauugAuaug....   1    1    JCK-3
.....................uaaaagauuugauggauuguuaug....   1    0    JCK-2
.....................Aaaaagauuugauggauuguuauga....  1    1    LT-3
......................aaaagauuugauggauug....        1    0    LT-2
......................aaaagauuugauggauug....        4    0    LT-3
......................aaaagauuugauggauugu....       2    0    JT-2
......................aaaagauuugauggauugu....       6    0    JT-3
......................aaaagauuugCuggauugu....       1    1    JT-2
......................aaaagauuugauggauuguu....      2    0    LT-1
......................aaaagauuugauggauuguu....      4    0    LT-3
......................aaaagauuugauggauuguC....      1    1    JCK-3
......................aaaagauuugauggauuguC....      1    1    JCK-2
......................aaaagauuCgauggauuguu....      1    1    LT-1
......................aGaagauuugauggauuguua....     6    1    JCK-1
......................aaaagauuugauggauuguua....     1    0    LT-3
......................aaaagauuugauggauuguuau....    4    0    JCK-2
......................aaaagauuugauggauuguuau....    1    0    LT-2
......................aaaagauuugauggauuguuau....    2    0    JCK-1
......................aaaagauuugauggauuguuau....    2    0    JCK-3
......................aaaagCuuugauggauuguuau....    1    1    JCK-3
......................aaGagauuugauggauuguuau....    1    1    JCK-3
......................aaaagauuugauggauuguuaug....   12   0    JT-3
......................aaaagauuugauggNuuguuaug....   1    1    LCK-1
......................aaaUgauuugauggauuguuaug....   2    1    JCK-2
......................aaaagauuugauggauuguuaug....   23   0    LCK-2
......................aaaagauuugauggauuguuaug....   4    0    LT-2
......................aaaUgauuugauggauuguuaug....   2    1    JCK-1
......................aaGagauuugauggauuguuaug....   2    1    JCK-2
......................aaaagauuugauggauuguuaug....   20   0    JT-1
......................aaaagauuugauggauuguuauA....   1    1    JCK-3
......................aaaagauuugauggauuguuaug....   17   0    LCK-1
......................aaaagauuugauggauuguuauC....   4    1    JCK-2
......................aaaUgauuugauggauuguuaug....   1    1    LT-3
......................aaaagauuugauggauuguuaug....   24   0    JCK-3
......................aaaagauuugauggauuguAaug....   7    1    LCK-3
......................aCaagauuugauggauuguuaug....   1    1    JCK-2
......................aaaagauuugauggauuguuaug....   6    0    LT-1
......................aaaagauuugauAgauuguuaug....   2    1    JCK-1
......................aaaagauuugauggauuguuGug....   2    1    JCK-3
......................aaaagauuugauggauuguuaug....   4    0    LCK-3
......................aaaagauuugauggauuguuaug....   20   0    JCK-2
......................aaaagauuugauggauuguuaug....   5    0    JT-2
......................aaaagauuugauggauugCuaug....   2    1    JCK-1
......................aaaagauuugauggauuguuGug....   1    1    JCK-1
......................aaaNgauuugauggauuguuaug....   1    1    JCK-1
```

Mature Star

Sequence			
uuuaaaaugaguuauaaacuuuaaaagauuugauggauuguuaugaaaucuuaguucauaaaacauuuauaaaaauccaauaaaaucuugaagugaagauuggauuucguauuuacaauuuucuucacaauuuuauuuuaaaauccuuucaaauauauuaaaauucauuauuuuuuaaaaucuuuuaaaauuaaugaccuuuugaauaccuauagauuuuaaaauaauuuuauaaaaauuauaauugaaua			
......................aaaagauuugauAgauuguuaug	2	1	LCK-3
......................aaaagauuugauggauuguuauU	2	1	LT-1
......................aaaagauuugauggauuguuauA	2	1	LT-1
......................aaaagauuugauggauuguAaug	2	1	JCK-3
......................aaaagauuugauggauuCuuaug	1	1	JCK-3
......................aaaagauuugauggauuguuGug	2	1	JCK-2
......................aaaagauuugauggauuguuaug	10	0	LT-3
......................aaaagauuugauggauugCuaug	1	1	JCK-2
......................aaaagauuugauggauuguuaug	27	0	JCK-1
......................aaaagauuugauggauuguuaugU	7	1	JCK-3
......................aaaagauuugauggauuAuuauga	3	1	LT-2
......................aaaagauuugauAgauuguuauga	2	1	LT-1
......................aaaagauuugauggauuAuuauga	4	1	JCK-1
......................aaaagauuugauggauuguuauga	14	0	JCK-3
......................aaaagauuugauggauuAuuauga	2	1	JCK-2
......................aaaagauuugauggauuAuuauga	1	1	LT-1
......................aaaUgauuugauggauuguuauga	4	1	JCK-3
......................aaaagauuugauggauuAuuauga	1	1	JCK-3
......................aaaagauuugaugAauuguuauga	1	1	LT-1
......................aaaagauuugauggauuguNauga	1	1	JCK-1
......................aaaagauuuAauggauuguuauga	2	1	JCK-3
......................aaaagauuugauggauuguuauga	16	0	JCK-1
......................aaaUgauuugauggauuguuauga	5	1	JCK-2
......................aaaagauuugauggauuguuauga	1	0	LT-2
......................aaaagauuugauggauuguuauga	32	0	JCK-2
......................aaaagauuuAauggauuguuauga	1	1	JCK-2
......................aaaagauuuAauggauuguuauga	1	1	JCK-1
......................aaaagauuugaugAauuguuauga	1	1	LT-3
......................aaaagauuugauggauuguuaugU	6	1	JCK-2
......................aaaagauuugauggauuguuauga	4	0	LT-1
......................aaaagauuugauggauuguuaugU	1	1	JCK-1
......................aaaagauuuAauggauuguuaugaa	2	1	JCK-1
.......................aaagauuugauggauuguu	1	0	JCK-1
.......................aaagauuugauggauuguuau	1	0	JCK-2
.......................aaagauuugauggauuguuauA	1	1	LT-2
.......................aGagauuugauggauuguuaug	2	1	LT-2
.......................aaagauuugauggauuguuauga	3	0	JCK-3
.......................aaagauuugauggauuguuauga	2	0	JCK-2
.......................aaaAauuugauggauuguuaugaa	1	1	JCK-2
.......................aaagauuugauggauuAuuaugaa	1	1	JCK-2
.......................aaagauuugauggUuuguuaugaa	1	1	JCK-3
.......................aaagauuugauggauuguuaugaa	1	0	JCK-3
.......................aaagauuugauggauuguuaugaa	1	0	JCK-2
........................aagauuugauggauuguuaug	2	0	LT-1
........................aagauuugauAgauuguuaugaaa	3	1	JCK-3
........................aagauuugauAgauuguuaugaaa	2	1	JCK-2
.........................agauuugauggauuguuaug	2	0	LT-3
.........................Ugauuugauggauuguuaug	1	1	LT-3
.........................agauuugauggauuguuaug	1	0	JCK-3
.........................Ugauuugauggauuguuaug	1	1	LT-1
.........................agauuugauggauuguuaug	1	0	JCK-2
.........................aAauuugauggauuguuauga	1	1	JCK-2
.........................Ugauuugauggauuguuauga	1	1	JCK-1
.........................Ugauuugauggauuguuauga	4	1	JCK-2
.........................Ugauuugauggauuguuauga	2	1	JCK-3
.........................Ugauuugauggauuguuaugaaau	1	1	JCK-3
..........................gauuugauggauuguuaug	1	0	JCK-3
..........................gauuugauggauuguuaug	1	0	JCK-1
..........................gauuugauggauuguuauga	2	0	JCK-1
..........................gauuugauggauuguuauga	1	0	JCK-3
..........................gauuCgauggauuguuauga	1	1	LT-2
...........................auuugauggauuguuaug	1	0	LT-2
...........................auuugauggauuguuaug	1	0	JCK-1
...........................auuugauggauuguuaug	3	0	LT-3
...........................auuugauggauuguuauU	1	1	LT-3
...........................auuugauggauuguuauga	4	0	LCK-3
...........................auuugauggauuguuauga	3	0	LT-3
...........................auuugauAgauuguuauga	1	1	LT-2
...........................auuugauggauuguuauga	6	0	JCK-2
...........................Uuuugauggauuguuauga	1	1	LT-3

```
                      Mature                              Star
uuuaaaaugaguuauaaacuuuaaaagauuugauggauuguuaugaaaucuuaguucauaaaacauuuauaaaaauccaauaaaaucuugaagugaagauuggauuucguauuuacaauuuucuucacaauuuuauuuuaaaauccuuucaaauauauuaaaauucauuauuuuuuaaaaucuuuuaaaauuaaugaccuuuugaauaccuauagauuuuaaaauaauuuuauaaaauuauaauugaaua
...........................auuugauggauuguuaAgaa...........................................................................................................................................................................................   1   1   LT-3
...........................auuugauggauuguuaugUa...........................................................................................................................................................................................   1   1   LT-3
...........................auuugauggauuguuaugaU...........................................................................................................................................................................................   1   1   JCK-3
...........................auuugauggauuguuaugaaa..........................................................................................................................................................................................   1   0   LT-1
...........................auuugauggauuguuaugaaau.........................................................................................................................................................................................   3   0   JCK-3
...........................auuugauggauuguuaugaaau.........................................................................................................................................................................................   1   0   JCK-1
...........................auuugauggauuguuauAaaau.........................................................................................................................................................................................   7   1   JT-3
...........................auuugauggauuguuaugaaau.........................................................................................................................................................................................   2   0   JCK-2
............................uuugauggauuguuauga............................................................................................................................................................................................   2   0   LT-1
............................uuugaAggauuguuauga............................................................................................................................................................................................   4   1   LT-2
............................uuugauggauuguuauUaa...........................................................................................................................................................................................   1   1   LT-2
............................uuugauggauuguuaugaa...........................................................................................................................................................................................   13  0   LCK-1
............................uuugauggauuguuaugaa...........................................................................................................................................................................................   5   0   LCK-2
............................uuugauggauuguuaugaaau.........................................................................................................................................................................................   1   0   JCK-1
..............................ugauggauuguAaugaaa..........................................................................................................................................................................................   6   1   JT-2
..............................ugauggauuguAaugaaa..........................................................................................................................................................................................   9   1   JT-3
...................................................................................................uuggauuucguauuGacaauuuuc...............................................................................................................................   2   1   JCK-2
...................................................................................................uuggauuucguauuGacaauuuuc...............................................................................................................................   2   1   JCK-1
............................................................................................................................................................................uuuuaaaaucuuuuCaaauu..........................................................   1   1   LT-3
...............................................................................................................................................................................................Caaugaccuuuugaauacc........................................   4   1   LT-2
...............................................................................................................................................................................................uaaugaccuuuugaauaccu.......................................   7   0   LCK-3
................................................................................................................................................................................................aaugaccuuuugaauacU........................................   1   1   JCK-2
................................................................................................................................................................................................aaugacUuuuugaauaccuauag...................................   9   1   LCK-3
................................................................................................................................................................................................aaugaccuuuugaauaccuaCaga..................................   2   1   LT-1
................................................................................................................................................................................................aaugacUuuuugaauaccuauaga..................................   1   1   JCK-1
................................................................................................................................................................................................aaugaccuuuugaauaccuauaga..................................   1   0   JCK-3
................................................................................................................................................................................................aaugaccuuuugaauaccuaCaga..................................   1   1   LT-3
................................................................................................................................................................................................aaugacUuuuugaauaccuauaga..................................   1   1   JCK-2
................................................................................................................................................................................................aaugaccuuuugaauaccuauaga..................................   1   0   JCK-1
................................................................................................................................................................................................aaugaccuuuugaauaccuauaga..................................   1   0   JCK-2
.................................................................................................................................................................................................augaccuuuugaauaccuaCagau.................................   1   1   LT-1
```
